# The Human Connectome: A Structural Description of the Human Brain

**DOI:** 10.1371/journal.pcbi.0010042

**Published:** 2005-09-30

**Authors:** Olaf Sporns, Giulio Tononi, Rolf Kötter

## Abstract

The connection matrix of the human brain (the human “connectome”) represents an indispensable foundation for basic and applied neurobiological research. However, the network of anatomical connections linking the neuronal elements of the human brain is still largely unknown. While some databases or collations of large-scale anatomical connection patterns exist for other mammalian species, there is currently no connection matrix of the human brain, nor is there a coordinated research effort to collect, archive, and disseminate this important information. We propose a research strategy to achieve this goal, and discuss its potential impact.

## Introduction

To understand the functioning of a network, one must know its elements and their interconnections. The purpose of this article is to discuss research strategies aimed at a comprehensive structural description of the network of elements and connections forming the human brain. We propose to call this dataset the human “connectome,” and we argue that it is fundamentally important in cognitive neuroscience and neuropsychology. The connectome will significantly increase our understanding of how functional brain states emerge from their underlying structural substrate, and will provide new mechanistic insights into how brain function is affected if this structural substrate is disrupted. It will provide a unified, time-invariant, and readily available neuroinformatics resource that could be used in virtually all areas of experimental and theoretical neuroscience.

Recent research in neuroscience has resulted in a rapid proliferation of neuroscience datasets and in the arrival of a new discipline, neuroinformatics [1–4]. Despite considerable advances in experimental techniques and computational paradigms, we still have an incomplete understanding of how human cognitive function emerges from neuronal structure and dynamics. Here, we focus on the relative lack of information about the different types of neural elements and their neural connections in the human brain. While a larger number of anatomical studies of the human brain have been carried out at the macroscopic (cerebral lobes, surface landmarks, and white matter tracts) or microscopic (cytoarchitectonics, myeloarchitectonics, chemoarchitectonics, etc.) anatomical level, there is virtually no information on the finer connectivity patterns, including neuronal connection densities or laminar projection patterns in relation to anatomically segregated cortical areas or intraregional differentiation. Furthermore, none of the available information is deposited in a single standardized data format, nor can it be accessed through a public database.

Experimental approaches to human cognition have been significantly enhanced by the arrival of functional neuroimaging [5], a set of techniques that can be applied to study a broad range of cognitive functions, with ever-increasing spatial and temporal resolution. But the mechanistic interpretation of neuroimaging data is limited, in part due to a severe lack of information on the structure and dynamics of the networks that generate the observed activation patterns. A potential theoretical framework for conceptualizing cognition as a network phenomenon is based on two main organizational principles found in the cerebral cortex, functional segregation, and functional integration [6,7]. Emerging network theories of cognition emphasize the contextual [8], distributed [9], dynamic [10], and degenerate [11,12] nature of structure–function mappings in the brain. To successfully map structure to function in the human brain, we urgently need a comprehensive, detailed structural model of neuronal units and their connections. Connectional models of the human brain are scarce and poorly defined [13], and they are largely based on extrapolating anatomical information from other primate species such as the macaque monkey, an approach that is problematic [14], in part, because of our incomplete understanding of evolutionary changes.

Our proposal to assemble the human connectome has several components. First, we attempt to define a level of scale at which a first draft of the human connectome might be assembled. We consider several potential experimental and neuroinformatics approaches for creating this first draft. We then discuss potential problems and limitations of the connectome, including the central issues of individual variability and development. Finally, we sketch out the potential impact of the connectome in computational and cognitive neuroscience.

## Scales and Levels of Structural Description

The human genome is composed of approximately 3 × 10^9^ base pairs, containing around 20,000–30,000 genes [15]. The compilation of the genome was aided by the fact that base pairs and genes are relatively straightforward choices as basic structural elements. The connectome will consist of two main descriptors defining its network architecture: neural elements and neural connections. Data fields for these elements specify superordinate or subordinate structures, a normalized position within a standard coordinate system, and additional parameters such as physiological/biophysical metadata that are crucial for specifying neural dynamics. The set of all *N* elements constitutes the columns (targets) and rows (sources) of an *N^2^*–*N* connection matrix A, whose *a_ij_* entries represent connections between individual elements *i* and *j*. In keeping with conventions adopted by other authors [16,17], confirmed absence of a connection is denoted by *a_ij_* = 0, while confirmed presence of a connection (irrespective of its strength or physiological characteristics) results in *a_ij_* = 1. Once a connection is confirmed to be present, its nonzero matrix element receives additional data entries cataloguing a range of structural and physiological parameters, such as fiber direction, connection density, strength, sign (excitatory/inhibitory), conduction delay, potential modulatory effects (voltage dependence), etc. The union of the binary connection matrix and connection-specific physiological data then results in a structural description that combines connection topology and biophysics.

Compiling the connectome faces two significant challenges not shared by other natural or technological networks. First, the human brain is a highly complex organ with a great number of structurally distinct, heterogeneous, yet interconnected components. Because a primary application of the connectome will likely be a structural substrate for understanding human cognitive function and interpreting neuroimaging studies, a first draft of the connectome may focus on the structural description of the corticothalamic system, including all regions of the cortex and their associated thalamic nuclei. Extensions of this first draft might include additional connected regions (striatum, cerebellum, etc.), with the ultimate aim of compiling the connectome of the whole brain.

A second challenge is that basic structural elements of the human brain, in terms of network nodes and connections, are difficult to define. Different kinds of structural descriptions could target at least three rather distinct levels of organization. At opposite ends of the scale are the level of single neurons and synapses (microscale) and the level of anatomically distinct brain regions and inter-regional pathways (macroscale). Between these two levels is the level of neuronal groups or populations (mesoscale). It is important to determine which level of description is the most appropriate for a first draft of the human connectome.

### **Microscale: Single Neurons and Synapses**

Attempting to assemble the human connectome at the level of single neurons is unrealistic and will remain infeasible at least in the near future. With single neurons as the basic element, the size of the connectome would be several orders of magnitude larger than that of the genome, comprising an estimated 10^11^ neurons, with 10^15^ connections between them (approximately 10^10^ neurons and 10^13^ connections in the cortex alone) [18,19]. Recording or tracing 10^15^ connections is not only technically impossible, it may also be unnecessary. While a genomic mutation in a single base pair can have dramatic consequences, alterations of single synapses or cells have not been shown to have similar macroscopic effects. Instead, there is overwhelming evidence that human cognitive functions depend on the activity and coactivity of large populations of neurons in distributed networks, including the corticothalamic system [20]. Furthermore, individual neurons and connections are subject to rapid plastic changes. These changes include synaptic weights as well as structural remodeling of dendritic spines and presynaptic boutons [21], possibly switching synaptic connections between large numbers of potential synaptic sites [22,23]. We suggest that the vast number, high variability, and fast dynamics of individual neurons and synapses render them inappropriate as basic elements for an initial draft of the connectome.

### **Macroscale: Brain Regions and Pathways**

An advantage of single neurons is that the elements themselves are relatively easily demarcated and well defined. In contrast, brain areas and neuronal populations are more difficult to delineate. No single universally accepted parcellation scheme currently exists for human brain regions (e.g., areas of the cerebral cortex), posing a significant obstacle to creating a unified resource such as the connectome. In the human cerebral cortex, neurons are arranged in an unknown number of anatomically distinct regions and areas, perhaps on the order of 100 [24] or more. Different subdivisions of the human brain (e.g., brain stem, thalamus, cerebellum, or cortex) may require different criteria for parcellation.

Nonetheless, anatomically distinct brain regions and inter-regional pathways represent perhaps the most feasible organizational level for compiling a first draft of the human connectome. Several neuroinformatics resources recording large-scale connection patterns in the cerebral cortex of various mammalian species already exist, for example, for most cortical regions of the macaque monkey [16,17,25], cat [26], and rat [27]. Computational analyses of these datasets have revealed a broad range of network characteristics [28], including the existence of clusters of brain regions [29], hierarchical organization [30,31], small-world attributes [32,33], distinct functional streams [34], motifs [35], and areal contributions to global network measures [36].

A broad range of experimental approaches exist at the macroscale. Cerebral white matter has traditionally been taken as a marker of the amount of connectivity within a cortical system. The relative contribution of cerebral white matter has increased throughout phylogeny to such an extent that its volume and metabolic requirements may present a limitation to further increases in connectional complexity [37]. The structural organization of white matter has been investigated by dissection, histological staining, degeneration methods, and axonal tracing [38]. Axonal tracing methods are the main basis for existing mammalian large-scale connection matrices [16,25–27], and their systematic compilation is currently being refined [17,39] to extract more sophisticated data, perhaps with the help of automated text analysis. In the human brain, postmortem tracing with carbocyanine dyes has provided details of connectivity within and between adjacent areas [40,41]. However, because of the slow transport and the length of fibers in the human brain, this method fails to reveal more remote connections. Another tracing method employs the in vivo detection by magnetic resonance imaging of high-contrast rare earth ions (e.g., manganese [42]) that have been injected into fiber tracts or inhaled, and taken up by neurons. The invasive nature and potential toxicity of the procedure makes it an unlikely candidate for human connectivity analyses.

Recent noninvasive imaging methods (diffusion tensor imaging [DTI] in its several variants, commonly followed by computational tractography) have been shown to produce results that are consistent with known pathways formed by major fiber tracts in the human brain [43–45], although there continues to be some limitations in data acquisition and processing algorithms [46]. To disambiguate signals produced by crossing or intersecting fibers, advances in diffusion imaging technology may allow the resolution of multiple axonal pathways within a single image voxel [47,48]. Despite the promise of diffusion imaging, a systematic atlas of DTI-based neuroanatomy, including probabilistic data gathered from individual brains, has not yet been produced, and the relationship among tensor fields, fiber tracts, and neuronal connections remains controversial. This controversy is likely to be resolved only through a combination of anatomical tracing techniques with noninvasive diffusion and functional imaging.

Perhaps the most promising avenue for compiling the human connectome originates from the notion that individual brain regions maintain individual connection profiles. What defines a segregated brain region is that all its structural elements share highly similar long-range connectivity patterns, and that these patterns are dissimilar between brain regions. These connectivity patterns determine the region's functional properties [49], and also allow their anatomical delineation and mapping. Diffusion imaging can be used to identify borders between cortical areas [50], most clearly on the basis of differences in long-range connections to the thalamus [51,52]. The idea that patterns of connectivity can be used to identify areal boundaries has also been tested in a combination of functional imaging and DTI/tractography in the human medial frontal cortex [53]. First, connection probabilities from voxels within the medial frontal cortex to all other voxels in the rest of the brain were obtained. Binarized connection patterns were then used to calculate a cross-correlation matrix, which was examined for the existence of distinct clusters of voxels with shared connection patterns. Such clusters were taken to represent anatomically segregated areas corresponding to human supplementary motor area and pre- supplementary motor area, respectively. Functional mapping revealed close correspondence between DTI and functional activation patterns. While this combined structural–functional approach is computationally intensive, nothing prevents its application to the entire corticothalamic system. We suggest that the correlated use of noninvasive structural and functional imaging methods offers the most promising experimental route toward the human connectome.

Structural connection data obtained using this combined methodology can in principle be validated by histological or tracing methods. Likely, no single method will turn out to be sufficiently powerful or comprehensive. A systematic application of sophisticated diffusion-weighted imaging combined with spatially registered high-resolution anatomical or spectroscopic imaging, regional activation, and coactivation data (e.g., electroencephalography, magnetoencephalography) obtained within the same individual subject, may offer the most feasible strategy for mapping the human connectome at the macroscale. This first draft of the human connectome would take the form of a probabilistic map of voxel-by-voxel connectivity embedded within standard coordinates containing approximately 10^4^–10^5^ elements and approximately 10^5^–10^7^ structural connections. It would not only provide information on the large-scale connection patterns within the corticothalamic system, but also on parcellation of human cortex into distinct areas based on a combination of structural and functional data in the same individual. Since this connectivity matrix is voxel-based, it can be cross-referenced with existing reference templates and with population-based brain atlases.

### **Mesoscale: Minicolumns and Their Connection Patterns**

A first draft of the corticothalamic connectome at the macroscale might provide a comprehensive dataset comprising several hundred brain regions and thousands of pathways, but it does not incorporate information on functional subdivisions or segregated subcircuits within each brain region. While such a macroscale description will provide many fundamental insights into the large-scale organization of human cortex, it is an insufficient basis for a complete understanding of the human brain's functional dynamics and information processing capacities. A further step in this direction involves the characterization of connection patterns among elementary processing units, corresponding to local populations of neurons such as cortical minicolumns. Mountcastle [54,55] originally proposed the cortical minicolumn as a basic functional unit of mammalian cerebral cortex. While details of minicolumn architecture are likely to vary across different brain regions [56–58], minicolumns generally contain approximately 80–100 neurons, spanning all cortical layers, with a diameter of approximately 30–50 μm [55]. Minicolumns may possess relatively stereotypic internal processing, and maintain generic patterns of inputs and outputs with minicolumns in other regions [56–59].

Recent studies have provided evidence for functionally specialized and precisely wired subnetworks of neurons coexisting within single cortical columns [60,61]. These studies have shown that cortical columns may contain segregated subnetworks, corresponding to minicolumns, which promote greater intracolumnar functional independence and informational heterogeneity. The members of such subnetworks are selectively interconnected with each other, indicating that connections within and between minicolumns follow more complex rules than simple random patterns or Gaussian (distance-dependent) connection profiles.

Minicolumns may be a sensible choice for neural elements at the mesoscale of the connectome because they may represent basic functional elements that are crucial for cortical information processing. While tracing or recording all minicolumns in an individual brain is still impossible, it may be feasible to collect data on minicolumn anatomy for selected brain regions, which can then be “fit” into the appropriate positions within the macroscale connection matrix. Functional imaging at columnar resolution has been carried out in animal experiments using high field strength [62], and may be facilitated in the future through selective imaging of fast and spatially precise capillary cerebral blood flow response components [63]. Especially important for determining functional responses of brain regions are connection patterns between each region's constituent elements. Axonal tracing methods have revealed specific patterns of horizontal connections between individual cells and cell populations within a brain region, which are often found to preferentially link cells with similar response characteristics [64,65], resulting in clustered intra-areal connectivity. Such patterns might also be accessible in the human brain [40], and regional variations in such patterns may provide important clues regarding the way in which information is processed within each region.

The axonal tracing approach delivers minicolumn maps for each distinct brain region, including information on their functional segregation and local (intra-areal) interconnectivity. While parcellated brain regions at the macroscale can be identified across individuals, we have no means to resolve the locations of corresponding minicolumns across different brains. The mapping of smaller-than-macroscopic units, therefore, requires coordinate-independent mapping approaches [66], which preserve the anatomical relationships and basic physiological properties of these units. Thus, macroscale and minicolumn descriptions deliver complementary datasets that need cross-level integration to achieve a single mesoscale version of the connectome. Effectively, minicolumn maps need to be mapped onto brain region–specific voxel sets rather than individual voxels, with voxel sets providing spatial embedding and probabilistic long-range connections, and minicolumn maps providing local connectivity, processing, and coding information. The minicolumn description provides a functionally heterogeneous architecture that is unique to each parcellated brain region, with specific (probabilistic) patterns of intra-areal and interareal minicolumn connections. A crucial task will be to convert long-range, nondirected voxel-by-voxel connectivity into directed, functionally heterogeneous long-range minicolumn interconnectivity. This requires intermediate descriptive levels such as stripes, bands, and blobs in the early visual system, for which specific connection patterns have been demonstrated across areal boundaries. When accomplished, this cross-level integration will result in a mesoscale connection matrix of the human brain that might comprise as many as 10^7^–10^8^ structural elements (comparable in size to the 2005 World Wide Web), with minicolumn elements directly cross-referenced to voxels in the macroscale map.

## Individual Variability and Development

The large-scale connectivity structure of the brain above the synaptic level represents a relatively invariant characteristic of our species. Once the elements and connections in the human brain are recorded, this dataset will remain stable, essentially forever. However, as in the case of the genome, the precise combinations and patterns of elements and their connections exhibit significant variations between individuals, at all levels of scale. Some individual variations may be due to genetic differences, others may be the result of developmental and experiential history, gender differences, pathologies, or responses to injury. To complicate matters further, the human connectome undergoes development through time, from early stages of the embryo to adolescence to adult age. Incorporating individual variations and developmental stages is absolutely crucial in rendering the connectome an effective resource.

Anatomical and imaging studies have revealed significant interindividual variability in the size and location of brain areas, as well as in the relationship between macrostructures (e.g., the cortical gyrification pattern) and microstructures (e.g., cytoarchitectonics and cytochemistry). Statistical analysis of variations in macroscopic surface features of the human brain, for example its sulcal geometry [67], demonstrates the extent to which even large-scale features of cortical morphology vary between individuals, possibly as a result of genetic differences [68]. An anatomical study of Broca's area in ten postmortem human brains revealed significant variations in size as well as in the area's relation to sulcal landmarks [69]. Applying structural magnetic resonance imaging to map the boundaries of the planum temporale has demonstrated significant variations in its size and position across 50 individuals [70]. Functional neuroimaging studies have also revealed significant interindividual differences, for example, in the location and extent of area MT/V5 [71] and other visual cortical areas [72]. These functional differences are presumably due to variations in underlying structural (cytoarchitectonic and connectional) substrates.

The presence of significant interindividual variability in structural connection patterns, even at the macroscale level, and the fundamentally probabilistic nature of connectivity datasets provided in the connectome may be viewed as fundamental weaknesses of the proposal, undermining its comprehensive goal of a definitive structural description of the human brain. However, we should consider the fact that there is also clear interindividual variability in the human genome. Nevertheless, the first draft with a DNA sequence obtained from cells from only a few individuals [15] has proven immensely useful for gaining insights into general organizational features of the human genome. Mapping of interindividual variability in the connectome is a necessary further step, but does not detract from the potential insights gained from a first draft that does not yet systematically incorporate these differences.

## Steps Toward the Human Connectome: Its Compilation, Assembly, and Integration

Based on a combination of functional and diffusion-weighted imaging, we outline the following five-step strategy for compiling a first draft of the human connectome at the macroscale.

Step 1 is to perform diffusion-weighted imaging followed by probabilistic tractography of thalamocortical tracts as well as corticocortical interareal pathways, using correlations in connectivity profiles to assist in parcellating human cortical regions. The end result is a voxel-wise probabilistic all-to-all structural connectivity matrix for the human brain. Step 2 is to perform a correlation analysis of spatially registered, equally resolved resting activity and/or multistimulus/multitask activation data (functional magnetic resonance imaging and/or magnetoencephalography) recorded in the same person [73], emphasizing strong functional relationships that are consistent across tasks [74]. The end result is a voxel-wise all-to-all functional connectivity matrix for the human brain. Step 3 is to perform a cluster analysis of correspondences between the structural and functional connectivity matrix obtained under steps 1 and 2, with the goal of identifying regions of consistent structure–function relationships in the human brain, possibly involving indirect projections [75]. Step 4 is to compare the results obtained by cluster analysis (step 3) with macaque data in order to identify correspondences (e.g., in visuomotor pathways) and deviations (e.g., in structures such as the fasciculus arcuatus). Step 5 is to validate the strongest predictions generated by assembling the final combined structural–functional connectivity matrix using custom-designed stimuli and perturbational techniques such as transcranial magnetic stimulation.

The following three steps represent additional stages designed to further test and verify the human connectome, including population analyses of individual variability and pathology. Step 6 is to perform a population analysis of healthy subjects and spatial registration to standard brain coordinates for probabilistic statements about data from steps 1–5. Step 7 is to compare population data on clustered brain regions to histologically identified regions in a probabilistic human brain atlas to assess correspondence. Step 8 is to compare population data between healthy individuals and patient groups with specific pathologies, to identify connectional differences.

Ultimately, the connectome will likely describe connectivity patterns at multiple levels of scale, for example, by incorporating linkages between the macroscale of brain regions and pathways in more elementary mesoscale functional units such as minicolumns and their patterns of interconnectivity. As experimental techniques mature, the connectome will gradually evolve through different stages of assembly as it is refined, updated, cross-validated, and “filled in.” Standardization of parcellation schemes, elimination of unreliable data, and incorporation of additional structural levels above and below the one chosen for the initial draft will drive this effort. An additional driving force is the continued innovation in data acquisition and analysis techniques, particularly in diffusion-weighted imaging and tractography, which will result in progressive revision, refinement, and extension of the connectome. To become a useful research tool, the connectome must be linked to other databases (compiled in parallel efforts) that record additional information mapped across the human brain, such as receptor distributions or gene expression patterns. We expect that assembling even the first draft of the connectome will require significant experimental and computational resources over an extended period of time. Compilation, assembly, and integration efforts are likely to be extensive tasks, requiring large-scale collaboration, coordinated data collection and dissemination, and the establishment of new computational methods, data standards, and mechanisms for controlled validation and quality assurance.

## Conclusions: The Potential Impact of the Connectome

How can the connectome be used to map brain structure to function? The step from structure to function is essential for understanding how cognitive processes emerge from their morphological substrates. Our central motivating hypothesis is that the pattern of elements and connections as captured in the connectome places specific constraints on brain dynamics, and thus shapes the operations and processes of human cognition. In turn, data recording the activity of the human brain in combination with the structural model provided by the connectome will help to discern causal interactions in large-scale brain networks (e.g., [76–78]). We emphasize that structure–function relationships are not directly evident from the connectional dataset itself. Rather, their elucidation will require further intense empirical and computational study. Depending on sensory input, global brain state, or learning, the same structural network can support a wide range of dynamic and cognitive states. This should not, however, discourage the effort to assemble the connectome. Similar difficulties in mapping structure to function exist in the case of the genome, although they generally were not foreseen when the project was initiated. Highly complex transcriptional regulatory networks, signaling pathways, mechanical forces, and elaborate mechanisms of gene expression all play essential roles in translating base sequences into functioning cells, tissues, and organisms. Both genome and connectome constitute complex networks [79], whose functions are not fully understood even if their structural substrates are fully catalogued.

An obvious and related use of the human connectome would be providing structural information that can be implemented as part of large-scale computational models [80,81]. If the connectome is sufficiently comprehensive and accessible, it could also provide a set of structural benchmarks that might facilitate the comparison and integration of the numerous specialized and structurally based models that have already been proposed in computational neuroscience. Drawing upon human connectional datasets would help ground modeling efforts aimed specifically at brain mechanisms of human cognitive function (e.g., language). Other computational applications of the connectome are topological analyses of network structure [28], perturbational studies aiming at mapping structural disruption to functional defects, and synthetic brain imaging [82–84].

The human connectome could potentially have a major impact on our understanding of brain damage and subsequent recovery. The effects of developmental variations or abnormalities, traumatic brain injury, or neurodegenerative disease can all be captured as specific structural variants of the human connectome. The functional consequences of network perturbations will allow a better understanding of structural causes of dysfunction, and may permit the design of strategies for recovery based on network analysis. Understanding the basic network causes of brain diseases may also open new avenues for therapy and prevention by harnessing inherent network mechanisms that ensure robustness and compensation.

There are many structural and functional aspects that the human connectome, as envisioned in this article, does not contain or address. For example, it does not explicitly capture or catalogue the rich variety of neuronal morphologies, the diversity of physiological and biochemical neural subtypes, glial cells, or brain vascularization. Its first draft does not capture synaptic plasticity and remodeling, nonsynaptic communication, hormonal regulation, or degenerative processes. We do not view these issues as shortcomings that fundamentally undermine the usefulness or potential impact of the connectome. Rather, they illustrate the open-ended, integrative nature of the proposed research effort. The connectome will represent a foundational resource and a central reference point for a broad range of specialized databases [85], thus making federating these databases more effective. Most importantly, the connectome will provide an important tool for mechanistic modeling and interpretation of human functional brain data. 

## Abbreviation

DTI: diffusion tensor imaging
